# Supplementary material for: Automatic distractor generation in multiple-choice questions: a systematic literature review
Source: PeerJ Comput Sci. 2024 Nov 13;10:e2441. doi: 10.7717/peerj-cs.2441 (PMC11623049; doi:10.7717/peerj-cs.2441)
Supplement: Supplemental Information 1 [file peerj-cs-10-2441-s001.docx]

**Table 4:**

**Data source list and its category and related type of questions**

| **Data Source** | **Count** |
| --- | --- |
| **Corpus** | **20** |
| closest-in-meaning   - [eng] Collocation Dictionary (Yusuf, Hidayah & Adji, 2023) - [eng] corpus and WordNet (Susanti, Iida & Tokunaga, 2015)   fill-in-the blank   - ESL Lounge2 corpus (Panda et al., 2022) - GloVe; Google Corpus N-Gram (Hill & Simha, 2016) - [eng] lecture slide (Chughtai et al., 2022) - online knowledge database (DBPedia; Wikipedia) (Alvarez & Baldassarri, 2018) - [tam] Tamil Vocabulary (Murugan & R, 2021) - [lao] vocabulary (Qiu et al., 2021) - web pages of fourteen Indian Leaders and eleven Indian social reformers (Das et al., 2019)   mathematics   - Eedi content repository (Feng et al., 2024)   mixed   - 30 labeled web pages from 10 websites (Foucher et al., 2022) | reading comprehension   - [por] DBPedia; Word2vec; Translated SQUAD (Oliveira et al., 2023) - [eng] Wikipedia (Rodriguez-Torrealba, Garcia-Lopez & Garcia-Cabot, 2022)   wh-questions   - ProcessBank corpus (Araki et al., 2016) - [eng] Websites (Wikipedia; EspnCricinfo; Yahoo! Cricket; CricBuzz; Cricwaves) (Patra & Saha, 2019)   unspecified   - [eng] Automated Student Assessment Prize (ASAP) (Shin, Guo & Gierl, 2019) - E-HowNet; Wikipedia (Chu, Chen & Lin, 2012) - [eng] GENIA; GENIA EVENT; BioInfer;4 YPD; Yapex;5 MIPS;6 WEB7 corpus and BioMed8 (Afzal & Mitkov, 2014) - Ted talk video script; WordNet (Huang et al., 2014) - [eng] textbook (Ch & Saha, 2023) |
| **Dataset** | **18** |
| fill-in-the blank   - [eng] CLOTH and RACE (Wang et al., 2023) - [eng] common wrong answers (CWA) (March, Perret & Hubbard, 2021)   mathematics   - mathematical reasoning dataset (Dave et al., 2021)   reading comprehension   - [eng] custom dataset based on RACE (Qiu, Wu & Fan, 2020) - [spa] DG-RACE; CosmosQA; and SciQ (De-Fitero-Dominguez et al., 2024; de-Fitero-Dominguez, Garcia-Cabot & Garcia-Lopez, 2024) - [eng] EQG-RACE (Dijkstra et al., 2022) - [eng] RACE (Chung, Chan & Fan, 2020; Guo, Wang & Guo, 2023; Shuai et al., 2023; Gao et al., 2019) - [eng] RACE; Cosmos QA (Xie et al., 2022) | - [eng] RACE; DREAM (Shuai et al., 2021) - [eng] RACE; RACE++ (Maurya & Desarkar, 2020) - [swe] SweQUAD-MC (Kalpakchi & Boye, 2021)   Visual Question Answering   - MC VQA dataset; Visual7W; and VQAv2 dataset (Ding et al., 2024) - Visual7w (Lu et al., 2022)   wh-questions   - NewsQuizQA (Lelkes, Tran & Yu, 2021) - [nld] televic dataset; wezooz dataset (Bitew et al., 2022) - USMLE dataset (Baldwin et al., 2022)   unspecified   - SciQ (Le Berre et al., 2022) |
| **Knowledge Base** | **12** |
| closest-in-meaning   - [eng] WordNet taxonomy; JACET8000 word list (Susanti et al., 2018)   fill-in-the blank   - [eng] Probase and Wordnet (Ren & Q. Zhu, 2021) - [tha] WordNet (Kwankajornkiet, Suchato & Punyabukkana, 2016) - WordNet; Word2Vec; Wikipedia (Kumar, Banchs & D’Haro, 2015)   mixed   - Candidates from Stem–Key pairs and Appendix; (b) Candidates from WordNet; (c) Candidates from Ontology (Kumar et al., 2023) - the HistOnto and SemQ ontologies (Al-Yahya, 2014) | Visual Question Answering   - Knowledge Base; Image Repository (Singh et al., 2019)   wh-questions   - [eng] WordNet; wiktionary; google search (Maheen et al., 2022)   unspecified   - [eng] domain and granular ontologies (Deepak et al., 2019) - [eng] educational Biology ontology (Stasaski & Hearst, 2017) - ontologies (E.V. & Kumar P., 2015) - UMLS and Freebase (Ha & Yaneva, 2018) |

The language id based on ISO 639 is marked with [###], for example [eng] for English data.
