# Supplementary material for: Automatic distractor generation in multiple-choice questions: a systematic literature review
Source: PeerJ Comput Sci. 2024 Nov 13;10:e2441. doi: 10.7717/peerj-cs.2441 (PMC11623049; doi:10.7717/peerj-cs.2441)
Supplement: Supplemental Information 2 [file peerj-cs-10-2441-s002.docx]

**Table 6:**

**Method list for different types of questions with its data source**

| **Method** | **Count** |
| --- | --- |
| **Linguistic** | **7** |
| closest-in-meaning   - [C] Collocation and POS (Yusuf, Hidayah & Adji, 2023)   fill-in-the blank   - [-] part of speech tagging; rule based (Nikolova, 2009) - [C] part of speech tagging; synonyms (Hill & Simha, 2016) - [C] random selection from vocabulary; homonymy via levenshtein distance between words in vocabulary; word2vec (Qiu et al., 2021) | reading comprehension   - [C] SpaCy; POS Tag; NER; NLTK Wordnet; DBPedia; (Oliveira et al., 2023)   unspecified   - [C] LDA Topic Modelling (Shin, Guo & Gierl, 2019) - [C] part of speech tagging; synonyms; hyponyms (Huang et al., 2014) |
| **Pattern matching** | **14** |
| closest-in-meaning   - [C] lexical hierarchy in WordNet; co-occuring word; POS tagging (Susanti, Iida & Tokunaga, 2015)   fill-in-the blank   - [C] pattern search (Das et al., 2019; Seyler, Yahya & Berberich, 2017) - [K] Probase and Wordnet (Ren & Q. Zhu, 2021) - [C] WordNet (Alvarez & Baldassarri, 2018) - [K] WordNet hypernyms and coordinates; google translates; synsets tree (Kwankajornkiet, Suchato & Punyabukkana, 2016)   mathematics   - [D] rule-based (Dave et al., 2021)   mixed   - [K] OWL ontology model manipulation (Al-Yahya, 2014) | unspecified   - [C] GENIA tagger (Afzal & Mitkov, 2014) - [K] granular ontologies and WordNet (Deepak et al., 2019) - [K] Ontologies (Stasaski & Hearst, 2017) - [K] ontologies and RNL (E.V. & Kumar P., 2015)   Visual Question Answering   - [K] Stanford Scene Graph Parser; Stanford-OpenIE API from Stanford CoreNLP (Singh et al., 2019)   wh-questions   - [C] event graph (Araki et al., 2016)   [K] WordNet; wiktionary; google search (Maheen et al., 2022) |
| **Statistical** | **37** |
| fill-in-the blank   - [D] pre-trained language model (PLM); (Wang et al., 2023) - [C] round trip machine translation (Panda et al., 2022) - [C] sense2vec (Chughtai et al., 2022) - [K] using vector representation of words (word2vec); language model probabilities and dice coefficients (Kumar, Banchs & D’Haro, 2015) - [C] Word embedding; spelling similarity suffix rules; lexicalized morphological transformation (Murugan & R, 2021)   mathematics   - [C] Large Language Model (GPT4) Prompting (Feng et al., 2024)   mixed   - [K] BERT; WordNet;ontology (Kumar et al., 2023) - [C] T5 closed book; entity similarity; sense2vec (Foucher et al., 2022)   reading comprehension   - [-] hierarchical encoder decoder framework with static and dynamic attention mechanisms (Gao et al., 2019) - [D] BERT (Chung, Chan & Fan, 2020; Kalpakchi & Boye, 2021) - [-] Deep Learning (Shuai et al., 2023) - [D] GPT-3 (Dijkstra et al., 2022) - [D] hierarchical encoder-decoder network with dynamic attention (Shuai et al., 2021) - [D] Hierarchical Multi-Decoder Network (HMD-Net) (LSTM) (Maurya & Desarkar, 2020) | - [D] mT5 (De-Fitero-Dominguez et al., 2024; de-Fitero-Dominguez, Garcia-Cabot & Garcia-Lopez, 2024) - [D] sequence-to-sequence based network (Qiu, Wu & Fan, 2020) - [D] T5; InstructGPT; BART (Guo, Wang & Guo, 2023)   unspecified   - [K] concept embeddings with information retrieval methods. (Ha & Yaneva, 2018) - [C] jaccard similarity; word embedding; structural information (Ch & Saha, 2023) - [-] Large Language Model Prompting (Grévisse, Pavlou & Schneider, 2024; Kıyak et al., 2024; Lin & Chen, 2024; Olney, 2023) - [D] RoBERTa (Le Berre et al., 2022) - [-] Sense2Vec; T5 (Saddish et al., 2023)   Visual Question Answering   - [D] Multi Layer Perceptron (MLP) (Lu et al., 2022) - [D] VL-T5 (Ding et al., 2024)   wh-questions   - [D] context-aware multilingual models (mBERT) (Bitew et al., 2022) - [D] cross-validated logistic regression model (Baldwin et al., 2022) - [C] statistical similarity; semantic similarity (Patra & Saha, 2019) - [D] T5 (Lelkes, Tran & Yu, 2021; Rodriguez-Torrealba, Garcia-Lopez & Garcia-Cabot, 2022; Xie et al., 2022) - [K] word2vec (Das et al., 2021; Murugan & Sadhu Ramakrishnan, 2022; Susanti et al., 2018) |

The data source is coded with Corpus as [C], Dataset as [D], Knowledge Base as [K], and unspecified as [-]
